# Neurotrophins Plasma Levels Kinetics in Ischemic Stroke Patients—Potential Relation to Outcomes

**DOI:** 10.3390/neurolint18030051

**Published:** 2026-03-04

**Authors:** Radosław Opiła, Karolina Łuczkowska, Edyta Paczkowska, Przemysław Nowacki, Jarosław Peregud-Pogorzelski, Bogusław Machaliński

**Affiliations:** 1 Department of Pediatrics, Oncology and Pediatric Immunology, Pomeranian Medical University, 71-252 Szczecin, Poland; 2 Department of General Pathology, Pomeranian Medical University, 70-111 Szczecin, Poland; 3 Department of Hematology and Bone Marrow Transplantation, Pomeranian Medical University, 71-252 Szczecin, Poland; 4 Neurology Department, Pomeranian Medical University, 71-252 Szczecin, Poland

**Keywords:** ischemic stroke, neurotrophins, NT3, NT4, BDNF, NGF

## Abstract

**Background/Objectives**: Neurotrophins are a family of structurally related growth factors known to play an important role in the physiology and pathophysiology of the central nervous system. In ischemic stroke, lower blood concentrations of brain-derived neurotrophic factor (BDNF) have been linked to worse outcomes. However, data regarding blood levels of other neurotrophins remain limited. **Methods**: Plasma levels of BDNF, NGF, NT-3 and NT-4 of 93 patients with ischemic stroke were measured using Luminex immunoassay at two time points: within 24 h from onset and on the seventh day. Clinical data regarding co-existing risk factors, National Institutes of Health Stroke Scale (NIHSS) score and mortality were collected and analyzed in relation to analytes. **Results**: BDNF levels at both time points were lower in patients with severe stroke and correlated negatively with NIHSS scores. No such associations were observed for NGF and NT-3. Patients who died had lower baseline BDNF, NT-4 and higher NT-3. **Conclusions**: A lower BDNF level, but no other neurotrophins, is associated with worse outcomes in ischemic stroke patients. NT-3 and NT-4 levels change in response to ischemic stroke.

## 1. Introduction

Neurotrophins (NTs) are a group of structurally related proteins involved in the development and regulation of the nervous system. They are considered a part of a broader group of peptides with similar roles, known as neurotrophic factors, which include substances with different structures and receptor specificities. Other groups of neurotrophic factors include the glial cell-lined-derived neurotrophic factor (GDNF) family, neuropoietic cytokines (including ciliary neurotrophic factor—CNTF), as well as other structurally and functionally distinct entities, for example, cerebral dopamine neurotrophic factor (CDNF) and mesencephalic astrocyte-derived neurotrophic factor (MANF), described in recent decades [1,2]. Inside the neurotrophin family, four main peptides, named classical NTs, have been described in humans: brain-derived neurotrophic factor (BDNF), nerve growth factor (NGF or beta-NGF), neurotrophin-3 (NT-3) and neurotrophin-4 (NT-4). Each of them exhibits varying affinity for their respective Trk receptors, the common p75 receptor and distinct expression patterns across the nervous system. Due to their physiological role in promoting survival and proliferation of neurons, enhancing synaptogenesis, and supporting regeneration after nerve injury, neurotrophins have been vigorously investigated for their potential biomarker and therapeutic properties [3]. BDNF was the one most widely studied, and its expression has been found to be altered in depression, dementia, schizophrenia, Parkinson’s disease, and epilepsy [4,5]. Experimental studies in animals and healthy human volunteers have shown that blood levels of BDNF correspond directly to its production and expression in the central nervous system, although a significant portion of circulating BDNF is of peripheral origin [3,6,7].

Unlike BDNF, there is no direct evidence confirming whether blood levels of NGF, NT-3 and NT-4 accurately reflect their production and expression in brain tissue. Some permeability of the blood–brain barrier (BBB) for NT-3, NGF and NT-4 was experimentally demonstrated in the blood-to-brain direction [8]. Studies linking blood alterations of those proteins to certain psychiatric and neurological diseases suggest a possible transfer in the opposite direction as well, at least under certain pathological conditions. Ischemic stroke is among the conditions known to disrupt the BBB [9]. Changes in circulating NGF, NT-3 and NT-4 were demonstrated in mood disorders, including bipolar disease and major depression [4,10,11]. NGF alterations were also confirmed in schizophrenia [12]. All neurotrophins can be produced and released in the bloodstream by peripheral tissues, and their levels can be affected by common non-neurological diseases (e.g., asthma and rheumatic diseases), which could limit their reliability as biomarkers of central nervous system pathology [13,14,15]. Nevertheless, since blood remains the most accessible material for clinical analysis, studies on plasma alterations of neurotrophins remain essential regardless of all their limitations.

Ischemic stroke is one of the leading causes of death and disability in developed countries. Despite its high prevalence, clinical data on neurotrophins in stroke remain very limited. Once again, BDNF was most extensively studied in this context. Meta-analysis conducted by Karantali et al. revealed that lower serum BDNF level measured on the first day after stroke are linked to worse outcomes and higher National Institutes of Health Stroke Scale (NIHSS) scores [16]. Lower BDNF levels in stroke patients were also associated with post-stroke depression [17]. Data considering other neurotrophins are scarce. In one study, the serum level of NGF collected on the first day after onset correlated negatively with NIHSS and modified Rankin Scale (mRS) assessed 3 months after diagnosis [18]. In a large proteomic profiling study conducted by Lagging et al., plasma NGF (measured in both the acute and convalescent phase) was among factors that were associated with better cognitive function seven years after stroke—unfortunately, no other neurotrophins were analyzed in this study [19]. Lower levels of NGF, similar to BDNF, were associated with post-stroke depression [20]. Regarding NT-3 and NT-4 in cerebral ischemia, articles published to date are mostly limited to animal studies [21,22]. Only one study measuring NT-3 levels in both serum and cerebrospinal fluid of stroke patients was published to date, though it focused on NT-3’s relation to IL-6, involved a small study group and did not provide comparison of NT-3 in serum and cerebrospinal fluid [23]. To our knowledge, no studies focusing on blood levels of NT-4 in human ischemic stroke were published.

In summary, the role of individual neurotrophins in response to cerebral ischemia remains uncertain. In this study, we aimed to investigate whether NGF, NT-3, NT-4 and BDNF plasma levels respond to ischemic stroke and how they relate to outcome.

## 2. Materials and Methods

### 2.1. Subjects

The study group consists of 93 patients who suffered from ischemic stroke and were admitted to the Department of Neurology, Pomeranian Medical University in Szczecin within 24 h from the first symptoms. We collected plasma samples from those patients at two time points—right after admission and on the seventh day of hospital care. Thirteen patients died, ten of them before the second time point. Two samples at the first time point and seven at the second time point were lost or destroyed during handling and, thus, excluded from analysis in respective time points. In summary, 91 eligible plasma samples on the first day, and 76 plasma samples on the seventh day were analyzed. NIHSS (National Institutes of Health Stroke Scale) score was attributed to patients at the end of hospital care.

We divided our subjects into two cohorts: 31 patients with severe stroke (those with an NIHSS score ≥ 16 and/or death during hospitalization) and 62 patients with mild-to-moderate stroke (details in Table 1, Supplementary File S1).

### 2.2. Laboratory Measurement Method

All blood samples were collected into EDTA anticoagulant tubes. Directly after collection, they were centrifuged for 10 min at 2000 rpm in order to separate the plasma. The plasma was then immediately frozen and kept in −80 °C until the assay was conducted.

Multiplex analysis of neurotrophic factors was carried out using Luminex-based bead technology. The assay relies on fluorescently coded magnetic microspheres, each population distinguished by a specific spectral profile and functionalized with capture molecules selective for individual targets. Quantification of NT-4, BDNF, NGF, and NT-3 was performed using the Human Luminex Discovery Assay (Bio-Techne, Minneapolis, MN, USA).

Plasma samples were used undiluted for the analysis. For each determination, equal volumes of plasma and bead suspension (25 µL each) were incubated in microplate wells for 2 h at room temperature with continuous shaking (800 rpm) and protected from light. After magnetic separation and triple washing with buffer, biotinylated detection antibodies were added and incubated for 1 h under the same conditions. Signal development was achieved by adding streptavidin conjugated to phycoerythrin and incubating for 30 min. Fluorescence signals were acquired using a Luminex 200 instrument (Luminex Corporation, Austin, TX, USA), and analyte concentrations were calculated from a seven-point standard curve.

### 2.3. Statistics

Statistical analysis was conducted with JASP, version 0.95.1 [24]. The Shapiro–Wilk test was used to determine the normality of data distribution, and outliers were identified with box plots. Mann–Whitney U test was used to compare two independent groups (Severe vs. Non-severe stroke). Wilcoxon signed-rank test was used to compare paired variables (Day 1 vs. Day 7). Correlations between continuous variables (neurotrophin levels, age and NIHSS scores) were tested with Spearman’s rank correlation coefficient. Missing values were excluded pairwise. Values under limit of detection were counted as 0 and excluded from analyses comparing paired variables (Day 1 vs. Day 7) and analyses of correlation with NIHSS. For additional analyses regarding NT-4, patients were divided into two groups representing baseline NT-4 below and above median. Groups were then compared using Mann–Whitney U test for difference in NIHSS and Chi-square test for severity and mortality.

All visualizations were created with JASP, version 0.95.1. A *p*-value of less than 0.05 was set for statistical significance.

## 3. Results

In the case of NT-4, many samples had readings below lower detection limit of the assay (0.08 pg/mL), limiting power and scope of the statistical analyses. Such “null” readings occurred in 38 samples (42%) from the first and 62 (82%) from the second time point. For BDNF, NT-3 and NGF, there were no samples exceeding either upper or lower detection limit, allowing standard statistical analyses to be performed.

**Figure 1:**
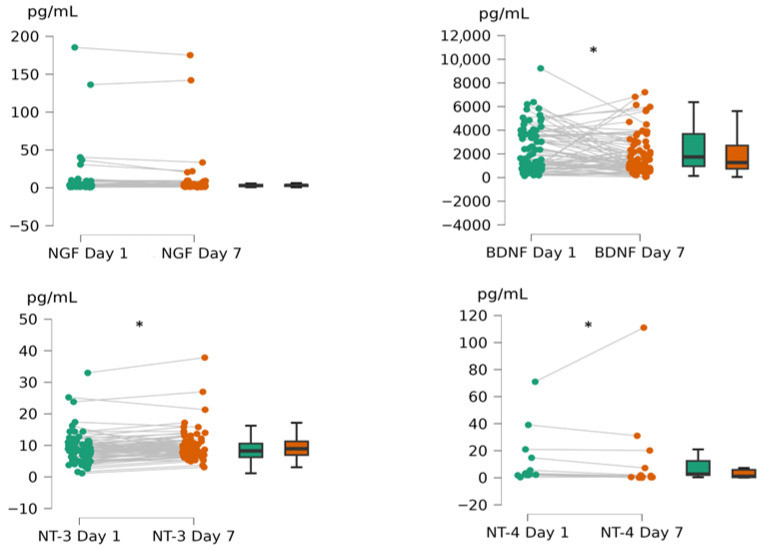
Change in neurotrophin levels over time (Day 1 vs. Day 7), visualized with scatter plot with points connected casewise and box plots, thick lines representing median values. Significant change (Day 7 compared to Day 1, *p* < 0.05) marked with *.

To assess how neurotrophins change over time in stroke, we compared their concentrations on Day 1 and Day 7. Significant change was observed for BDNF (r = 0.332, SE = 0.133, *p* = 0.013), NT-3 (r = −0.504, SE = 0.133, *p* < 0.001) and NT-4 (r = 0.714, SE = 0.294, *p* = 0.017), whereas NGF remained stable. BDNF and NT-4 decreased, while NT-3 increased over time (Figure 1).

**Figure 2:**
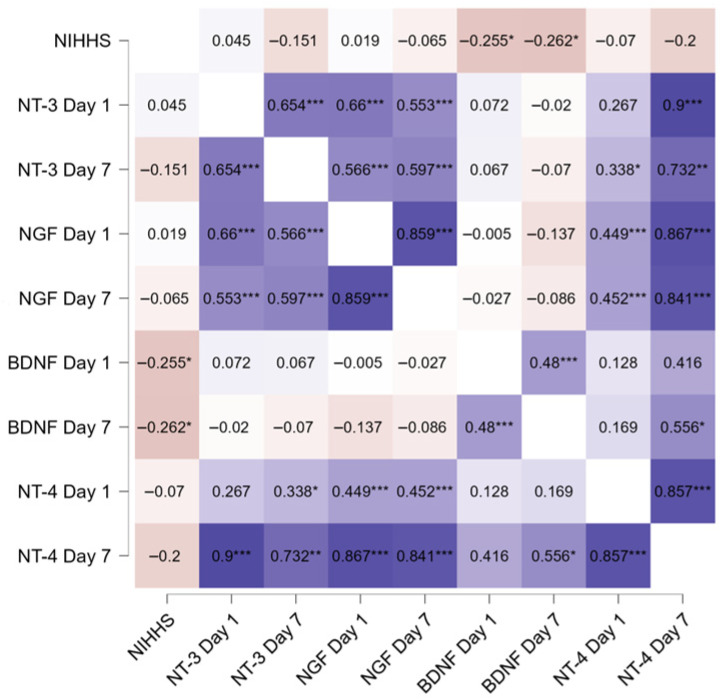
Neurotrophins and their correlation with NIHSS and each other—visualized with heatmap. Values represent Spearman’s rank correlation coefficient. *p* values: * <0.05 ** <0.01 *** <0.001.

**Figure 3:**
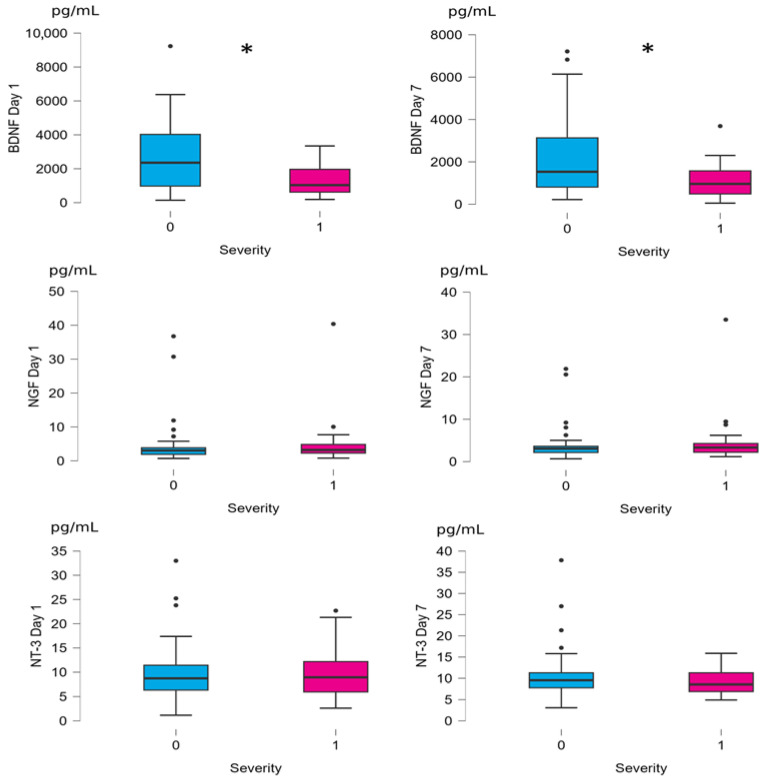
Differences between analyte concentrations between non-severe (0 on x-axis) and severe (1 on x-axis) stroke groups, box and whisker plot. Values on y-axis represent analyte concentration in pg/mL. Significant differences (severe stroke group compared to non-severe stroke group, *p* < 0.05) marked with *.

In the second step of our analysis, we examined potential associations of neurotrophins and clinical outcomes. BDNF correlated negatively with NIHSS score at both time points, but no such correlation was found for NT-3 nor NGF (Table 2, Figure 2). Patients with severe stroke (NIHSS > 15 and/or death) had significantly lower BDNF at both time points, while no differences were observed in regard to NT-3 and NGF (Table 3, Figure 3). BDNF level did not correlate directly with any other neurotrophin, but NT-3, NT-4 and NGF levels exhibited strong and positive correlations with each other at both time points (Figure 2). Patients who died during observation (*n* = 13) had significantly lower baseline BDNF (r = −0.353, SE = 0.172, *p* = 0.043) and higher NT-3 (r = 0.474, SE = 0.172, *p* = 0.006) than the rest of the group (*n* = 80). All 13 patients who died during observation had baseline NT-4 readings below the median, including twelve with values under limit of detection of the method used (aforementioned “null” readings). Patients with baseline NT-4 levels below and above the median did not differ significantly in NIHSS scores or in the proportion of patients with severe stroke.

Patients who received tissue plasminogen activator treatment (tPA; *n* = 19) did not differ significantly from the rest of the group in neurotrophin levels at either time point. However, no observable change in neurotrophin over time was observed in this subset of patients. This might reflect small sample size or a result of specific time window (within 4 h since onset of symptoms), in which those samples were collected, but might also suggest a treatment-related effect on stabilizing neurotrophin profile.

Diabetes mellitus was associated with lower BDNF at the second time point (r = −0.421, SE = 0.144, *p* = 0.003), but presence of other established stroke risk factors (smoking, atrial fibrillation, alcoholism, arterial hypertension, dyslipidemia, obesity) did not present any significant associations with analyte concentrations. There was also no significant difference in analyte levels between males and females and no significant correlation with age. However, there were significant differences in age, sex, and distribution of risk factors between severe and non-severe stroke groups. There were more females, infections and fewer tobacco smokers in the severe stroke group. Also, patients with severe stroke tended to be older (Table 1). When adjusted for those factors, BDNF was still significantly lower at both time points in the severe stroke group (Table A1).

Among patients who died during hospital care (*n* = 13), there was a significantly greater share of females (X^2^ = 5.050; *p* = 0.025), more infections (X^2^ = 8.450; *p* = 0.004), and atrial fibrillation (X^2^ = 4.382; *p* = 0.036). When adjusted for these factors, findings regarding baseline NT-3 and BDNF association with mortality remained significant (Table A2).

NIHSS was positively correlated with age and higher in females and patients presenting with infection or atrial fibrillation. When adjusted for these factors, correlation with BDNF at both time points remained significant (Table A3).

## 4. Discussion

Current knowledge about role of individual neurotrophins in the regeneration of mature human brain damage and ischemia focuses heavily on BDNF and NGF. Most studies on central nervous system damage of various etiologies (including stroke and traumatic brain injury—TBI) concentrate on those two peptides [3]. Both BDNF and NGF have been proposed as promising therapeutic agents in brain injury and stroke. Less is known about NT-3 and NT-4 in this context. Both have been shown to alter their expression in response to brain injury in animal models, but no direct human data is available [25]. They both promote survival of retinal neurons when administered after optic nerve injury in rats, and NT-3 has been evaluated as a promising therapeutic agent in spinal cord and peripheral nerve injury [26,27,28,29]. Establishing their role in ischemic stroke might provide a crucial step in search for novel therapeutic agents. Our study provides novel insights into plasma concentrations of NT-3 and NT-4 in patients with acute ischemic stroke.

Our results regarding BDNF overlap with already existing research on this neurotrophin. We confirmed its negative correlation with NIHSS and showed an association with outcome, as described in earlier studies [16]. Despite the BDNF level change between days 1 and 7, these associations remained significant at both time points. Together with previously reported data associating lower BDNF level with overall higher risk of stroke, this implies that the pre-existing status of BDNF in the brain influences its post-stroke measure and, possibly, its relation to outcomes [30]. This is especially relevant from a clinical perspective, as BDNF levels have previously been shown to depend on such modifiable factors as anaerobic physical exercise, dyslipidemia, and diabetes [31,32,33].

Unlike Luan et al., our study did not establish any relation between NGF and outcome, nor did we observe any significant change in NGF over time [18]. Several differences in study design could account for this discrepancy. First, there is a notable difference in sample size. Second, we assessed the outcome at different time points. Lack of NGF change between time points in our study indicates only that it remains stable over the first 7 days after stroke, but does not exclude very early or late alterations of its level in response to cerebral ischemia.

In our study, the observed changes in NT-3 and NT-4 levels within the first seven days after stroke—evident both in comparisons of paired variables and in the striking disproportion of values below the detection limit between the two time points (in the case of NT-4)—suggest that stroke alters plasma NT-3 and NT-4 levels. NT-3 increased over time, and higher baseline concentrations were observed in patients who died. This suggest increase in NT-3 expression in response to stroke. Whether this effect is due to peripheral or CNS NT-3 expression remains an open question. Unlike BDNF, NT-3 did not show any significant correlation with NIHSS or severe outcome; thus, NT-3 is not necessarily a marker of stroke severity, despite its connection with mortality. Aside from possible statistical bias, higher NT-3 in patients who died might have been influenced by its expression in peripheral tissues due to potential severe comorbidities. NT-3 is abundant in peripheral tissues, and its expression depends on sympathetic innervation in animal studies [34,35]. Sympathetic excitation in response to stress in critical illness might elevate NT-3 levels in patients who died, without any significant link to neurological outcome.

Opposed to NT-3, NT-4 decreased over time, and its baseline concentrations were lower in patients who died. This indicates either lower expression of NT-4 in response to stroke or its increased depletion. NT-4 is expressed in skeletal muscles, and this expression decreases in response to axonal damage in animal models [36,37]. Sudden loss of efferent signaling in stroke-related paresis might then explain the decrease in plasma NT-4. If that were true, the NT-4 decrease could relate to the extent of post-stroke paresis; however, testing for such a hypothesis lies beyond the limits of our study. Our data set does not allow proper analysis on NT-4 relation with NIHSS. Strong, mutual correlations of NT-3, NGF and NT-4—but not BDNF—may suggest a shared regulatory background in the baseline expression of the three, while underlining the distinct role of BDNF in response to ischemia.

Several limitations of our study have to be taken into account when discussing the results. First, there was no healthy control group to match the results of ischemic stroke patients. This approach allowed us to recruit more participants into the study but eliminated the possibility of directly verifying whether neurotrophin levels differ in stroke patients compared to healthy individuals. Moreover, given the short half-life of neurotrophins, this introduces uncertainty as to whether the second or the first time point is closer to the pre-ischemia state. Second, we must remember the large disproportion in group size between patients who died vs. survivors, which limits the statistical power of results linking NT-3 and NT-4 with mortality, especially since we did not find any significant results linking NT-3 or NT-4 levels with outcomes. In particular, results regarding NT-4 should be considered rough and preliminary due to the structure of our data set (high percentage of results below the detection limit). While it may be insufficient to draw any strong conclusions, it could encourage further analysis, deploying more sensitive methods or an improved study design. Third, as there are no direct data indicating linear correlation of NGF, NT-4, and NT-3 expression in CNS and their blood concentrations, our results might not illustrate genuine neurotrophin dynamics in situ. More experimental research is necessary to address this issue.

## 5. Conclusions

Among all measured neurotrophins, BDNF showed the most pronounced association with outcome in the acute phase after stroke. Plasma BDNF correlated negatively with NIHSS score at Day 1 and Day 7 and was significantly lower in patients with severe stroke and those who died during hospital care. Plasma NT-3 baseline level is higher in patients who died during observation, but no other associations with outcome could be drawn from our results. There were no evident associations of plasma NGF and outcome in the acute phase of stroke. NT-4 levels below the median were associated with higher mortality, although no direct correlation with NIHSS or severity could be demonstrated. Plasma BDNF and NT-4 decreased within the first seven days after stroke, while NT-3 increased slightly and NGF remains stable. Beyond already well-established alterations in BDNF in stroke, our findings suggest that NT-3 and NT-4 levels also respond to ischemic stroke, showing a faint association with mortality in its acute phase.

## Figures and Tables

**Table 1:** Demographic and clinical characteristics of study group.

	Total Subjects (*n*= 93)	Non-Severe (*n* = 62)	Severe ^†^ (*n* = 31)	Χ^2^	*p*
Age, median (range, years) **	77 (34–92)	74.5 (34–92)	79 (41–92)	-	0.023 **
Sex, male/female *	52/41 (56%/44%)	33/29 (53%/47%)	8/23 (26%/74%)	6.303	0.012 *
Activated plasminogen treatment	19 (20%)	12 (19%)	7 (23%)	0.132	0.716
Tobacco *	43 (46%)	33 (53%)	10 (32%)	4.394	0.036 *
Hypertension	79 (85%)	51 (82%)	28 (90%)	1.051	0.305
Diabetes	33 (35%)	18 (29%)	15 (48%)	3.185	0.074
Overweight/Obesity	54 (58%)	36 (58%)	18 (58%)	0.053	0.819
Alcohol abuse	22 (24%)	14 (23%)	8 (26%)	0.122	0.727
Hypercholesterolemia	55 (59%)	36 (58%)	19 (61%)	0.089	0.765
Hypertriglyceridemia	20 (21%)	13 (21%)	7 (23%)	0.032	0.858
Atrial fibrillation *	27 (29%)	11 (18%)	16 (52%)	11.18	<0.001 *
History of stroke/myocardial infarction myocardial infarction	30 (32%)	19 (31%)	11 (35%)	0.135	0.714
Infection	31 (33%)	20 (32%)	11 (35%)	4.429	0.035 *
NIHSS score, median (range) **	7 (0–30)	5 (0–15)	18.5 (5–30)	-	<001 **

Values are presented as medians (interquartile range) and absolute numbers (percentage, rounded); * significant (*p* < 0.05) difference between groups, measured with Chi-square test (for nominal variables); ** significant (*p* < 0.05) difference between groups, measured with Mann–Whitney U test (age); ^†^ severe stroke group defined as patients with NIHHS score ≥ 16 and/or death during hospitalization.

**Table 2:** Correlation of analytes with NIHSS (The National Institutes of Health Stroke Scale).

		Spearman’s Rank Correlation Coefficient
**Day 1**	BDNF	−0.255 *
	NGF	0.019
	NT-3	0.045
	NT-4	−0.070
**Day 7**	BDNF	−0.262 *
	NGF	−0.065
	NT-3	−0.151
	NT-4	−0.200

* *p* < 0.05.

**Table 3:** Comparison of analyte levels between non-severe and severe stroke groups.

	All	Non-Severe	Severe	U	*p*
BDNF Day 1	1584 (140.2–9232)	2355 (140.3–9232)	1034 (188.0–3344)	1278.0	0.004 *
BDNF Day 7	1268 (53.07–7213)	1533 (222.3–7213)	962 (53.07–3690)	759.0	0.019 *
NGF Day 1	3.210 (0.720–185.5)	3.135 (0.720–185.5)	3.210 (0.81–40.39)	859.0	0.555
NGF Day 7	3.300 (0.660–175.2))	3.185 (0.66–175.2)	3.325 (1.160–33.49)	531.5	0.741
NT-3 Day 1	8.930 (1.160–32.99)	8.720 (1.160–32.99)	8.930 (2.620–22.69)	889.0	0.734
NT-3 Day 7	9.230 (3.080–37.83)	9.520 (3.080–37.83)	8.580(4.920–15.87)	644.5	0.321
NT-4 Day 1	0.925 (0–71.0)	1.226 (0–71.00)	0.000 (0–5.526)	-	-
NT-4 Day 7	0.000 (0–111.0)	0.000 (0–111.0)	0.000 (0–1.855)	-	-

Mann–Whitney U test was performed to establish difference between non-severe and severe stroke groups. Severe stroke group defined as final NIHSS above or equal to 16 and/or death during observation. Values represent median (range). All concentrations are in pg/mL. * *p* < 0.05.

## Data Availability

The original contributions presented in this study are included in the article/Supplementary Material. Further inquiries can be directed to the corresponding authors.

## Supplementary Materials

The following supporting information can be downloaded at: https://www.mdpi.com/article/10.3390/neurolint18030051/s1, Supplementary File S1: Database.

## Abbreviations

The following abbreviations are used in this manuscript:

NIHSS	National Institutes of Health Stroke Scale
NT	Neurotrophins
BBB	Blood–brain barrier
CNS	Central nervous system

## Appendix A

This appendix contains tables regarding additional multivariate analyses.

**Table A1:** Relation of BDNF levels and stroke severity adjusted for potential confounding factors.

	Logistic Regression Model					
	Estimate	Standard Error	Odds Ratio	z	Wald Statistic	*p*
**BDNF Day 1**	−0.001	0.000	0.999	−2.985	8.907	0.003 *
Atrial fibrillation	1.812	0.700	6.122	2.587	6.695	0.010 *
Infection	0.081	0.680	1.085	0.120	0.014	0.905
Sex	0.512	0.727	1.669	0.705	0.497	0.481
Nicotinism	0.695	0.688	2.004	1.010	1.020	0.313
Age	−0.015	0.028	0.985	−0.536	0.287	0.592
**BDNF Day 7**	−0.001	0.000	0.999	−2.267	5.139	0.023 *
Atrial fibrillation	1.511	0.773	4.531	1.955	3.821	0.051
Infection	−0.345	0.808	0.708	−0.427	0.182	0.669
Sex	0.685	0.757	1.983	0.905	0.819	0.365
Nicotinism	0.277	0.795	1.320	0.349	0.122	0.727
Age	0.010	0.032	1.010	0.322	0.104	0.747

Logistic regression model. Stroke severity (severe vs. non-severe stroke) treated as dependent variable. Severe stroke group defined as final NIHSS above or equal to 16 and/or death during observation. * *p* < 0.05.

**Table A2:** Relation of BDNF and NT-3 levels and mortality (death vs. no death during observation) adjusted for potential confounding factors.

	Univariate		Logistic Regression Model					
	U	*p*	Estimate	Standard Error	Odds Ratio	z	Wald Statistic	*p*
**BDNF Day 1**	686.00	0.043 *	−0.001	0.000	0.999	−2.077	4.314	0.038 *
Atrial fibrillation			0.609	0.731	1.839	0.834	0.695	0.404
Infection			1.146	0.729	3.146	1.572	2.470	0.116
Sex			1.293	0.867	3.642	1.491	2.223	0.136
**NT-3 Day 1**	266.50	0.006 *	0.128	0.055	1.136	2.319	5.376	0.020 *
Atrial fibrillation			0.353	0.720	1.423	0.490	0.240	0.624
Infection			1.415	0.735	4.117	1.926	3.708	0.054
Sex			1.134	0.894	3.108	1.268	1.607	0.205
**NGF Day 1**	350.00	0.076	-	-	-	-	-	-

Logistic regression model. Death during observation (death vs. no death) treated as dependent variable. * *p* < 0.05.

**Table A3:** Correlation of NIHHS and BDNF, adjusted for potential confounding factors.

	Generalized Linear Model			
	Estimate	Standard Error	t	*p*
Sex	1.646	1.854	0.888	0.377
Atrial fibrillation	3.815	1.913	1.994	0.049 *
Infection	1.098	2.111	0.520	0.604
Age	0.103	0.077	1.334	0.186
**BDNF Day 1**	−0.001	4.228 × 10^−4^	−3.143	0.002 *
Sex	0.859	1.986	0.433	0.667
Atrial fibrillation	2.710	2.073	1.307	0.195
Infection	0.239	2.402	0.100	0.921
Age	0.117	0.078	1.499	0.138
**BDNF Day 7**	−0.001	4.981 × 10^−4^	−2.125	0.037 *

Generalized Linear Model using Gaussian identity link. NIHSS treated as dependent variable. * *p* < 0.05.
